# Comment: difference between assessment of upper limb movement and upper limb associated reactions during walking

**DOI:** 10.1186/s12984-021-00844-0

**Published:** 2021-03-10

**Authors:** Pieter Meyns

**Affiliations:** REVAL (Rehabilitation Research), Faculty of Rehabilitation Sciences, Hasselt University Campus Diepenbeek, Agoralaan gebouw D, B-3590 Hasselt, Belgium

**Keywords:** Gait, Arm swing, Coordination, Spasticity, Kinematics, Arm posture

## Abstract

**Background:**

While walking, people swing their arms in a specific pattern. This specific arm swing pattern during walking has shown to have a beneficial effect on gait as it reduces walking energy cost and optimizes balance. In several patient populations the arm movements can be directly affected (e.g. in patients with acquired brain injury (ABI)), which in turn has a negative effect on their gait pattern, balance and energy cost of walking.

**Main text:**

In December 2019, Kahn et al. published a paper in JNER concerning the quantification of upper limb associated reactions (ARs) during walking in people with ABI. ARs are defined as “an effort-dependent phenomenon causing an involuntary increase in upper limb muscle tone, with awkward and uncomfortable postures”. These upper limb ARs appear often in patients with ABI and can have an important effect on their gait. The authors calculated kinematic measures using three-dimensional gait analysis relating to range of motion, variability and mean position over the gait cycle for the different upper limb joints (shoulder, elbow, wrist) during self-selected steady-state walking. Based on differences they found between an ABI cohort and healthy control cohort, the authors concluded that they were able to quantify ARs during walking in this population. This calculation, however, is not specific for upper limb ARs. In fact, the authors calculated general measures of arm posture (e.g. mean position over the gait cycle) or arm movement (e.g. range of motion and variability) during gait. Previous research has already indicated that other factors than ARs can influence the posture or movement of the arm during gait in patients with brain injury, such as voluntary compensations for gait instability and contractures or spasticity of upper arm muscles. Yet, it is not possible to disentangle the different causes of the altered arm posture during steady-state walking based on the proposed measures.

**Conclusion:**

The kinematic arm measures proposed by Kahn et al. (J Neuroeng Rehabil 16(1):160, 2019) are not a direct measure of ARs, but provide a quantification of overall deviation of arm posture or movement during gait. Depending on the specific study design these measures may provide insights in ARs.

## Background

While walking, people swing their arms. At self-selected or preferred walking speeds, this arm swing in healthy adults shows a typical pattern as it is coordinated with the leg movements; while the left leg (right) swings forward, the right (left) arm swings forward [1]. At first sight, this arm swing appears a meaningless and irrelevant by-product of movements of the trunk which are passively transferred to the arms. Previous research, however, has shown that the arm movements during gait are not entirely passive, but are partly active to achieve this specific coordination with the legs [2]. Such specific “normal” coordinated arm swing has shown to reduce walking energy cost and have a positive effect on balance [1]. The arm swing or arm posture during gait can be quantified using movement analysis, and summarized in kinematic outcome parameters. As such, these kinematic outcome parameters are a direct reflection of the arm movement pattern. In patients with central neurologic pathologies, such as stroke, cerebral palsy and Parkinson’s disease, different symptoms (e.g. spasticity) can affect or cause the altered arm movement patterns, which in turn has an effect on the coordination between the arms and legs during gait and as such influences the gait pattern, balance and energy cost of walking [1].

## Main text

In December 2019, Kahn et al. published a paper in JNER concerning the quantification of upper limb associated reactions (ARs] during walking in people with acquired brain injury [3]. In their study, the authors have defined ARs as “an effort-dependent phenomenon causing an involuntary increase in upper limb muscle tone, with awkward and uncomfortable postures”. These ARs appear often and can impede the gait pattern, balance and walking energy cost in patients with acquired brain injury (ABI). This is a very relevant topic, worthy of investigation as quantification of this phenomenon allows to assess its effect on gait and can influence rehabilitation programs to address this issue to improve gait in these patients. In their paper, Kahn et al. assessed whether it was possible to quantify and assess ARs during walking from joint kinematics measured using three-dimensional motion analysis. The authors calculated measures relating to range of motion, variability (i.e. standard deviation) and mean position over the gait cycle for the different joints (shoulder, elbow, wrist) during self-selected steady-state walking and compared these to a healthy control cohort. Based on the differences between the ABI cohort and the healthy control cohort, the authors concluded that they were able to quantify ARs during walking in this population. This calculation, however, is not specific for upper limb ARs. In fact, the authors calculated kinematic measures of arm posture (e.g. mean position over the gait cycle) or arm movement (e.g. range of motion and variability) during gait, which are an overall reflection of the movement pattern. The authors, in this case, seem to assume that their kinematic arm measures in patients with ABI during gait are a direct reflection of the ARs, but the proposed kinematic measures cannot distinguish between the different causes or influencing factors of the altered movement pattern.

Previous research has already indicated that other factors (than ARs) can influence the posture or movement of the arm during gait in patients with brain injury as well. Children with cerebral palsy, for instance, have been shown to alter their arm movements during walking as a result of increased gait instability [4, 5]. In CP, the arm posture shows similarities to those described in toddlers that recently learned to walk (i.e. the elbow is more flexed and the hand is held in a high position e.g. above the pelvis), but contrary to ARs, this arm posture not necessarily unwanted or involuntary as it has been shown to be a solution to fulfill the requirements of postural stability and forward propulsion [6]. Furthermore, previous research in patients with stroke has indicated that spasticity affects the upper limb position (i.e. clinically the described with a flexed elbow, flexed wrist and closed fist) and lower limb position (i.e. usually the knee extensor muscles are involved resulting in a stiff knee, as are the ankle plantar-flexor muscles resulting in an equinovarus foot) when evaluated in isolation [7, 8]. The upper limb spasticity also affects the altered arm posture during gait, as Botulinum-toxin treatment of spastic upper arm muscles improves their arm swing movements and, consequently, their gait pattern [9, 10].

This means that an altered score on the kinematic arm measure quantified by Kahn et al. [3] is not necessarily an indication of ARs, but does show abnormal upper limb posture or movement during gait. For instance, if a patient has an elbow flexion contracture, which would already be visible when standing still, the patient will also show increased elbow flexion during walking which is not an AR. Similarly, it could be possible that a patient increases trunk and arm movements or adopts a specific arm posture to compensate for increased gait instability. These compensatory movements are then not directly related to the effort of walking, but are wanted/voluntary and, thus, do not align with the definition of ARs.

On the other hand, the phenomenon of ARs is scarcely investigated and is a relevant field of study. It may be possible to assess ARs using the proposed kinematic arm measures by Kahn et al. [3] if investigators create a study design which increases the effort of the patient without increasing his walking speed (as this may increase the velocity-dependent spasticity in these patients) and without changing the stability constraints of the walking condition (as this may influence gait stability). In this way, the proposed kinematic arm measures, which actually measure abnormal arm movement patterns, can be used to specifically assess ARs.

## Conclusions

In conclusion, the kinematic arm measures proposed by Kahn et al. [3] are not a direct measure of ARs, but provide a quantification of overall deviation of arm posture or movement during gait in patients with ABI. Depending on the specific study design such measures may provide insights in ARs in different populations in future studies.

## Data Availability

Not applicable.

## Abbreviations

ABI: Acquired brain injury
AR(s): Associated reaction(s)
